# Supplementary material for: Graphene: Hexagonal Boron Nitride Composite Films with Low-Resistance for Flexible Electronics
Source: Nanomaterials (Basel). 2022 May 17;12(10):1703. doi: 10.3390/nano12101703 (PMC9147634; doi:10.3390/nano12101703)
Supplement: Supplementary file 1 [file nanomaterials-12-01703-s001.zip › nanomaterials-1651444-supplementary.pdf]

nanomaterials

Supplementary Materials

Graphene: Hexagonal Boron Nitride Composite Films with Low-Resistance for Flexible Electronics

Irina V. Antonova [1,2,*], Marina B. Shavelkina [3], Artem I. Ivanov [1], Dmitriy A. Poteryaev [1,2], Nadezhda A. Nebogatikova [1], Anna A. Buzmakova [2], Regina A. Soots [1] and Vladimir A. Katarzhis [3]

1 Rzhanov Institute of Semiconductor Physics SB RAS, 13 Lavrentiev aven., Novosibirsk 630090, Russia; art.iv.il@mail.ru (A.I.I.); poteryayevd@inbox.ru (D.A.P.); nadonebo@gmail.com (N.A.N.); soots@isp.nsc.ru (R.A.S.)
2 Department of Semiconductor Devices and Microelectronics of Novosibirsk State Technical University, 20 K. Marx str., Novosibirsk630073, Russia; kiraromanova011@gmail.com (A.A.B.)
3 Joint Institute for High Temperatures RAS, Izhorskaya Str. 13 Bd.2, Moscow 125412, Russia; mshavelkina@gmail.com (M.B.S.); korg983@yandex.ru (V.A.K.)
* Correspondence: antonova@isp.nsc.ru; Tel: +7-913-731-2788

Three movies are attached as supplementary materials. The movies demonstrate the clustering process during the drying of the drops of different suspensions on SiO_2/Si substrates. The liquid component of suspensions in all cases consists of 70% ethanol and 30% deionized water. The fast process of the flake migration corresponds to the evaporation of the ethanol from the drop. After that, a slow clustering process was observed. Movies show the clustering (fast stage) in drops of the h-BN suspension, graphene suspension and composite h-BN : G suspension with content 1:1. In the case of the h-BN suspension, there are no large clusters at all. Drying of the graphene suspension leads to the formation of loose graphene clusters. The combination of the polar h-BN flakes with graphene strongly enhanced the clustering and led to the formation of dense h-BN : G clusters with self-organization effects (see Figures S1 and S2, and the movie h-BN-G). Addition of PEDOT:PSS to the h-BN : G suspension does not lead to a visible change in the clustering process. In the case of inks, the clustering process was limited by used additives and ultrasonic treatments.

Figure S3 demonstrates the scale of all movies and figures.

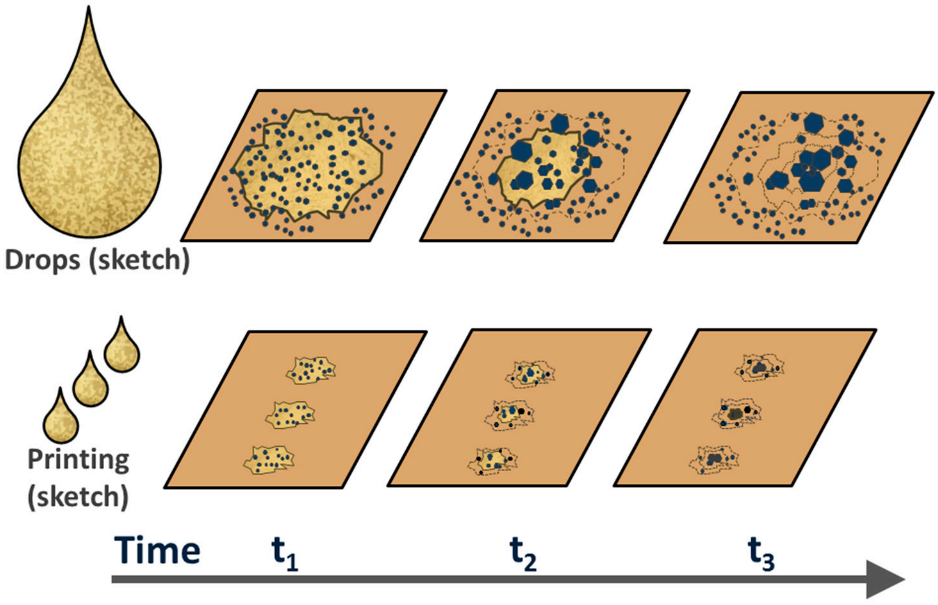

Figure S1. Schematic images of composite cluster formation for cases of drop using 2D printing.

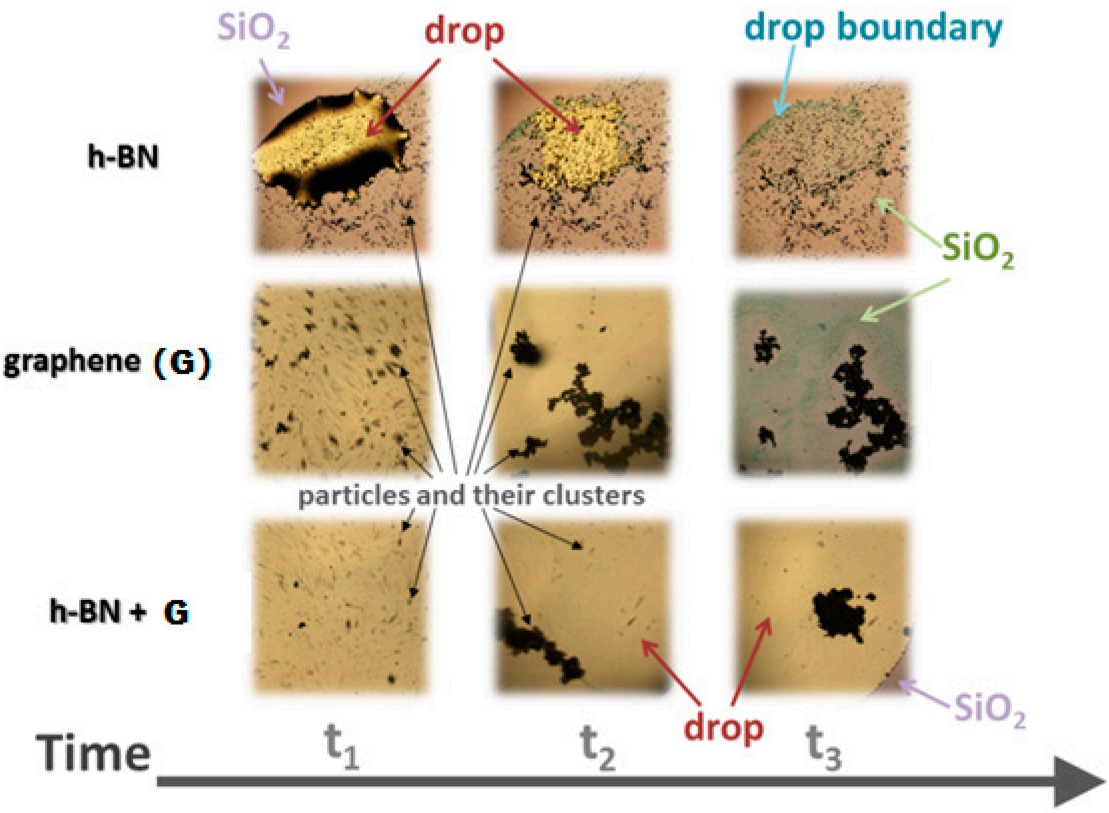

Figure S2. Optical microscopy images of components and composite cluster formation in the drops on SiO_2/Si substrates.

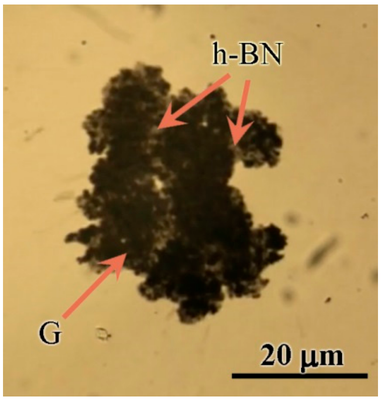

Figure S3. Optical microscopy image of graphene : h-BN cluster after drop drying on SiO_2/Si substrates.

As follows from the films and Figure 3, h-BN particles stimulate the clustering process and lead to the formation of the composite nanoparticle in the inks. The h-BN inclusions are seen as lighter color veins in the cluster.
